# The recovery rate from severe acute malnutrition among under-five years of children remains low in sub-Saharan Africa. A systematic review and meta-analysis of observational studies

**DOI:** 10.1371/journal.pone.0229698

**Published:** 2020-03-18

**Authors:** Hanna Demelash Desyibelew, Mulat Tirfie Bayih, Adhanom Gebreegziabher Baraki, Abel Fekadu Dadi

**Affiliations:** 1 Department of Public Health Nutrition, School of Public Health, College of Medicine and Health Sciences, Bahir Dar University, Bahir Dar, Ethiopia; 2 Department of Epidemiology and Biostatistics, College of Medicine and Health Sciences, Institute of Public Health, University of Gondar, Gondar, Ethiopia; 3 College of Medicine and Public Health, School of Public Health, Flinders University, Adelaide, Australia; San Raffaele Roma Open University, ITALY

## Abstract

**Background:**

Globally, Severe Acute Malnutrition (SAM) has been reduced by only 11% over the past 20 years and continues to be a significant cause of morbidity and mortality. So far, in Sub-Saharan Africa, several primary studies have been conducted on recovery rate and determinants of recovery from SAM in under-five children. However, comprehensive reviews that would have a shred of strong evidence for designing interventions are lacking. So, this review and meta-analysis was conducted to bridge this gap.

**Methods:**

A systematic review of observational studies published in the years between 1/1/2000 to 12/31/2018 was conducted following the Meta-analysis of Observational Studies in Epidemiology (MOOSE) statement. Two reviewers have been searched and extracted data from CINAHL (EBSCO), MEDLINE (via Ovid), Emcare, PubMed databases, and Google scholar. Articles' quality was assessed using the Newcastle-Ottawa Scale by two independent reviewers, and only studies with fair to good quality were included in the final analysis. The review presented the pooled recovery rate from SAM and an odds ratio of risk factors affecting recovery rate after checking for heterogeneity and publication bias. The review has been registered in PROSPERO with protocol number CRD42019122085.

**Result:**

Children with SAM from 54 primary studies (n = 140,148) were included. A pooled rate of recovery was 71.2% (95% CI: 68.5–73.8; *I*^*2*^ = 98.9%). Children who received routine medication (Pooled Odds ratio (POR):1.85;95% CI: 1.49–2.29; *I*^*2*^ = 0.0%), older age (POR: 1.99;95% CI: 1.29–3.08; *I*^*2*^ = 80.6%), and absence of co-morbidity (POR:3.2;95% CI: 2.15–4.76; *I*^*2*^ = 78.7%) had better odds of recovery. This systematic review and meta-analysis suggestes HIV infected children had lower recovery rate from SAM (POR; 0.19; 95% CI: 0.09–0.39; *I*^*2*^ = 42.9%) compared to those non-infected.

**Conclusion:**

The meta-analysis deciphers that the pooled recovery rate was below the SPHERE standard, and further works would be needed to improve the recovery rate. So, factors that were identified might help to revise the plan set by the countries, and further research might be required to explore health fascilities fidelity to the WHO SAM management protocol.

## Background

Severe Acute Malnutrition (SAM) is defined as weight-for-height/weight-for-length Z-score (WHZ) of <−3SD or an absolute mid-upper-arm circumference (MUAC) of < 115 mm with bilateral nutritional oedema [1]. Estimates revealed that globally numbers of children who suffer from acute malnutrition had been reduced very gradually (only 11% over the past 20 years), particularly when compared with the progress made in reducing other nutritional indicators like stunting [2].

According to the latest United Nations Children's Fund (UNICEF), World Health Organization (WHO), and World Bank (WB) joint child malnutrition estimates of 2018, 16.4 million under-five children worldwide (2.4%) are suffering from severe acute malnutrition. Of these, 2.2 million were found in Sub-Saharan African countries [3]. SAM is continuing to be the major public health problem and an essential contributor to morbidity and mortality in under-five children [4,5]. In interventions taken by community health workers, a recovery rate of around 90% was estimated in a systematic review and meta-analysis done in middle-and low-income counties [6]. A study done in Asian and African countries reported that recovery rates from SAM ranges from 25% to 95% at inpatient management and 50% to 93% at outpatient management program [7–12]. In Ethiopia, the estimated recovery rate from SAM in under-five children ranged from 67% to 88.4% at inpatient management program [13–18].

Although there were many primary studies conducted on the recovery rate of SAM in under-five children in Sub-saharan countries, informative and comprehensive reviews are lacking. Therefore, this review synthesized and summarized recovery rates and its associated factors taken from several independent primary studies to support intervention programs and to inform policy and practices in Sub-Saharan Africa [19].

## Methods and analysis

### Protocol design and registration

A systematic review and meta-analysis of observational studies published in the years from 1/1/2000 to 12/31/2018 was conducted. The Preferred Reporting Items for Systematic Reviews and Meta-analyses Protocol (PRISMA-P) [20,21], and Meta-analysis of Observational Studies in Epidemiology (MOOSE) guideline statement [22] were used for the development of this study protocol, the conduct and design, and the reporting of these results. To minimize duplication of the same reviews, provide transparency, and to reduce reporting bias of the current review, protocol was registered with the International Registration of Systematic Reviews (PROSPERO) with PROSPERO registration number CRD42019122085.

### Eligibility criteria

#### Inclusion criteria

Studies that used observational epidemiological designs (cross-sectional, case-control, prospective and retrospective cohort), involving severe acute malnutrition in under-five years of age children that assessed SAM using anthropometric screening tools and nutritional edema were included. Severe acute malnutrition was defined as weight-for-height z-score (WHZ) <-3 SD, weight-for-height (WFH) <70% of the median National Center for Health Statistics (NCHS) or WHO reference or mid-upper arm circumference (MUAC) <115mm. Articles published between January 1/2000 to January 31/2018 time period were considered for review, which we believed that sufficient time frame was considered for a demonstrable trend of under-five children SAM recovery rate.

#### Outcome definitions

Studies that only used the following definition for recovery were included in the review: children reaching >85% of the weight for height or weight for length or adding 15% of their admission weight and no edema for consecutive 4 days at the discharge of the severe acute malnutrition child.

#### Exclusion criteria

The review excluded studies conducted in high or low-risk SAM children such as those living in refugee camps as prevalence studies conducted in such a restricted population might not represent the general population. Besides, studies published other than the English language were omitted from the final analysis. Finally, eligible studies used unclear definition of severe acute malnutrition (like unclear measurement tools, admission, and discharge criteria) were excluded.

### Data sources and search strategies

The primary outcome of this review was the recovery rate of children with severe acute malnutrition. A search strategy has been developed using fundamental concepts in the research question: anthropometric assessment, SAM recovery rate, and Sub-Saharan Africa. For each key concept, appropriate free-text words and Medical Subject Headings (MeSH) were used and combined using boolean operators such as AND and OR. This enabled the retrieval of relevant articles that might have used different synonyms for the same word.

A pretest of the search strategy by two authors was performed in PubMed, and the actual electronic search was done between June 10 and 15, 2019. Two independent reviewers implemented the electronic search in the following electronic databases: CINAHL (EBSCO), MEDLINE (via Ovid), Emcare, PubMed, and Google scholar search engines. Moreove, snowballing and retrieving references or hand searching was performed on the reference lists of eligible studies to include studies that were unable to identify by the search strategy. Finally, the search process was presented in a PRISMA flow chart.

#### Example of search strategy in PubMed

(severe acute malnutrition recovery rate) OR severe acute malnutrition cure rate) OR nutritional recovery rate) OR severe acute malnutrition treatment outcome) OR protein-energy malnutrition) OR malnutrition) OR wasting)) AND ((under-five children) OR childhood): filters: Observational Study; Publication date from 2000/01/01 to 2018/12/31; Humans; English.

### Study selection

All citations identified by our search strategy, which were potentially eligible for inclusion, were exported to EndNote version 8, and duplicates were removed. Titles and abstracts of the remaining citations were screened by two independent reviewers and ineligible studies were further excluded. The full texts of selected articles were retrieved and read thoroughly to ascertain their suitability before data extraction.

### Data extraction process and quality assessment

The abstract and full-text review and data abstraction were done by two independent reviewers (HDD and AFD) using a standardized data abstraction form, developed according to the sequence of variables required from the primary studies on MS-Excel sheet. Disagreements in data abstraction between the two independent reviewers were resolved by a third independent reviewer (MTB or AGB). Before analysis, a transformation of Odds ratios and prevalence was made.

The Newcastle-Ottawa Scale (NOS) was selected for assessing the quality of the included studies. The NOS included 3 categorical criteria with a maximum score of 9 points. The quality of each study was rated using the following scoring algorithms: ≥7 points were considered as "good," 2 to 6 points were considered as "fair," and ≤ 1 point was considered as "poor" quality study. Accordingly, to maintain the validity of this systematic review, we only included primary studies with fair to good quality. The name of authors, year of publication, country, study setup, treatment center, sample size, MUAC, recovery rate prevalence, and risk factors associated with recovery rate variables were extracted. (Table 1)

**Table 1:** Characteristics of included study in meta-analysis of studies conducted on under-five children recovery rate in Sub-Saharan African countries (N = 54).

Authors,year	Country	Treatment setup	Treatment center	Sample size	Residence	MUAC cutoffs	Recovered	NOS
Admasu A, et al 2017	Ethiopia	Inpatient	HT	340	Urban-rural	11.5	75.6	7
ADAL TG, et al2016	Ethiopia	Inpatient	HT	450	Urban-rural	11.5	76.4	8
Akparibo R, et al, 2017	Ghana	OTP	All	488	Urban-rural	11.5	71	7
Amthor RE, et al 2009	Malawi	OTP	HC HP	826	Urban-rural	11	93.7	6
Asres DT,et al 2018	Ethiopia	Inpatient	HT	401	Urban-rural	11	51	7
Attia S, et al 2016	Malawi	Inpatient	HT	79	Urban-rural	11.5	77	6
Belachew T, et al 2007	Ethiopia	OTP	HC HP	1088	Urban-rural	11	51	6
Berti A, et al 2008	Ethiopia	Inpatient	HT	493	Urban-rural	11	88.4	6
Binns PJ, et al 2016	Malawi	OTP	HC HP	258	Urban-rural	11.5	63.2	6
Chane T, et al 2014	Ethiopia	Inpatient	HT	324	Urban-rural	11	85	7
Chiabi A, et al 2016	Cameroon	Inpatient	HT	106	Urban-rural	11.5	84	7
Chitekwe S, et al 2018	Nigeria	OTP	HC HP	102,245	Urban-rural	11.5	87.1	7
Dale NM, et al 2013	Sudan	OTP	HC	753	Urban-rural	11.5	82	7
Derseh B, et al 2018	Ethiopia	Inpatient	HT	413	Urban-rural	11	55.9	7
Desta K. et al 2015	Ethiopia	Inpatient	HC HP	415	Urban-rural	11	47	8
Desyibelew HD, et al 2017	Ethiopia	Inpatient	HT	401	Urban-rural	11	58.4	7
Eklund M, et al 2008	Ethiopia	OTP	HC	324	Urban-rural	--	45	5
Gaboulaud V, et al 2006	Niger	Inpatient	HT	1937	Urban-rural	11	74.5	8
Gebremichael DY. et al 2015	Ethiopia	Inpatient	HP	420	Urban-rural	11	82.4	7
Girum T, et al 2017	Ethiopia	Inpatient	HC HT	545	Urban-rural	11.5	76	8
Girum T, et al 2018	Ethiopia	Inpatient	HC HT	400	Urban-rural	11.5	75	8
Guesh G, et al 2018	Ethiopia	Inpatient	HT	569	Urban-rural	11.5	82	8
Irena AH, et al 2011	Zambia	Inpatient	HT	430	Urban-rural	--	53.7	4
Jarso H, et al 2015	Ethiopia	Inpatient	HT	947	Urban-rural	11	77.8	7
Kabalo MY, et al 2018	Ethiopia	Inpatient	HT	582	Urban-rural	11.5	68	8
Kabalo MY, *et al*. 2017	Ethiopia	OTP	HP	794	Urban-rural	11	64.9	7
Kabeta A, et al 2017	Ethiopia	Inpatient	HT	196	Urban-rural	11	78	8
Kanan SO, et al 2016	Sudan	Inpatient	HT	593	Urban-rural	--	75.7	7
Linneman Z, et al 2007	Malawi	OTP	HC	2131	Rural	--	89	7
Massa D, et al 2016	Ethiopia	OTP	HC	332	Rural	11	76.8	7
Mbaya D, et al 2017	Kenya	OTP	HT	104	Urban-rural	11.5	73.3	8
Mekuria G, et al 2017	Ethiopia	Inpatient	HT	253	Urban-rural	11	77.9	8
Mena MB, et al 2018	Ethiopia	Inpatient	HT	205	Urban-rural	11	66.8	8
Mengesha MM, et al. 2016	Ethiopia	OTP	HP	348	Urban-rural	11	78.7	8
Mumbere M, et al 2018	DRC	Inpatient	HT	136	Urban-rural	11.5	97	6
Muzigaba M, et al 2017	South Africa	Inpatient	HT	454	Rural	--	75.6	7
Ndzo JA, et al 2018	Cameroon	OTP	HT	254	Urban-rural	11.5	72.8	8
Nyeko R, et al 2016	Uganda	Inpatient	HT	251	Urban-rural	11.5	66.9	8
Okinyi LK. Et al 2018	Kenya	Inpatient	HT	160	Urban-rural	11.5	65	5
Oumer A, et al 2016	Ethiopia	Inpatient	HT	617	Urban-rural	11.5	69.9	8
Saaka M, et al 2015	Ghana	OTP	HC HT	348	Urban-rural	11.5	33.6	7
Sadler K, et al 2008	Malawi	Inpatient	HT	1077	Urban-rural	--	58.1	6
Shanka N, et al 2015	Ethiopia	OTP	HC HP	711	Urban-rural	11	67.7	8
Shiferaw W, et al 2015	Ethiopia	Inpatient	HT	151	Urban-rural	11	70	7
Teferi E, et al 2010	Ethiopia	Inpatient	HC	8485	Urban-rural	11	92	7
Tekeste A, et al 2012	Ethiopia	Both	HC	649	Urban-rural	--	94.3	5
Terefe Abeje A. et al 2016	Ethiopia	Inpatient	HT	298	Urban-rural	11.5	68.5	8
Teshome G, et al 2019	Ethiopia	OTP	HP	2216	Rural	11	79.6	7
Tirore MG, et al 2017	Ethiopia	Inpatient	HT	195	Urban-rural	11	43.6	8
Trehan I, et al 2010	Malawi	OTP	HC	2453	Rural	11	85	7
Ubesie AC, et al 2012	Nigeria	Inpatient	HT	212	Urban-rural	--	58.5	8
Wagnew F, et al 2018	Ethiopia	Inpatient	HT	527	Urban-rural	11.5	67.7	8
Wammanda R, et al 2002	Nigeria	Inpatient	HT	136	Urban-rural	--	38.9	5
Yebyo HG, et al 2013	Ethiopia	OTP	HC HP	628	Urban-rural	11	61.78	8

OTP: Out-Patient, MUAC: Mid Upper Arm Circumference, NOS: New-castle Ottaw Scale, HT: Hospital, HC: Health Center, HP: Health Post

## Data analysis

### Testing for heterogeneity

Heterogeneity between the results of the primary studies was assessed using the Cochran's Q test and quantified with the *I*^*2*^ statistic. A P-value of less than 0.1 was considered to suggest statistically significant heterogeneity, considering small number of studies and their heteregeinity in design [23]. Heterogeneity had taken low, moderate, and high categories when the I^2^ values were below 25%, between 25% and 75%, and above 75%, respectively [24]. Thus, the random effect model was used to pool the SAM recovery rate since the studies were found heterogeneous. The random effect model accounts for heterogeneity among study results beyond the variation associated with chance unlike fixed-effect model [25]. To investigate the sources of heterogeneity, the random-effects meta-regression was conducted by taking primary study characteristics such as study setting, study treatment program, MUAC admission cutoffs, and study location. The meta-regression analysis was weighted to account for the residual between-study heterogeneity (i.e., heterogeneity not explained by the covariates in the regression) [26].

### Publication bias assessment

**Fig 1:**
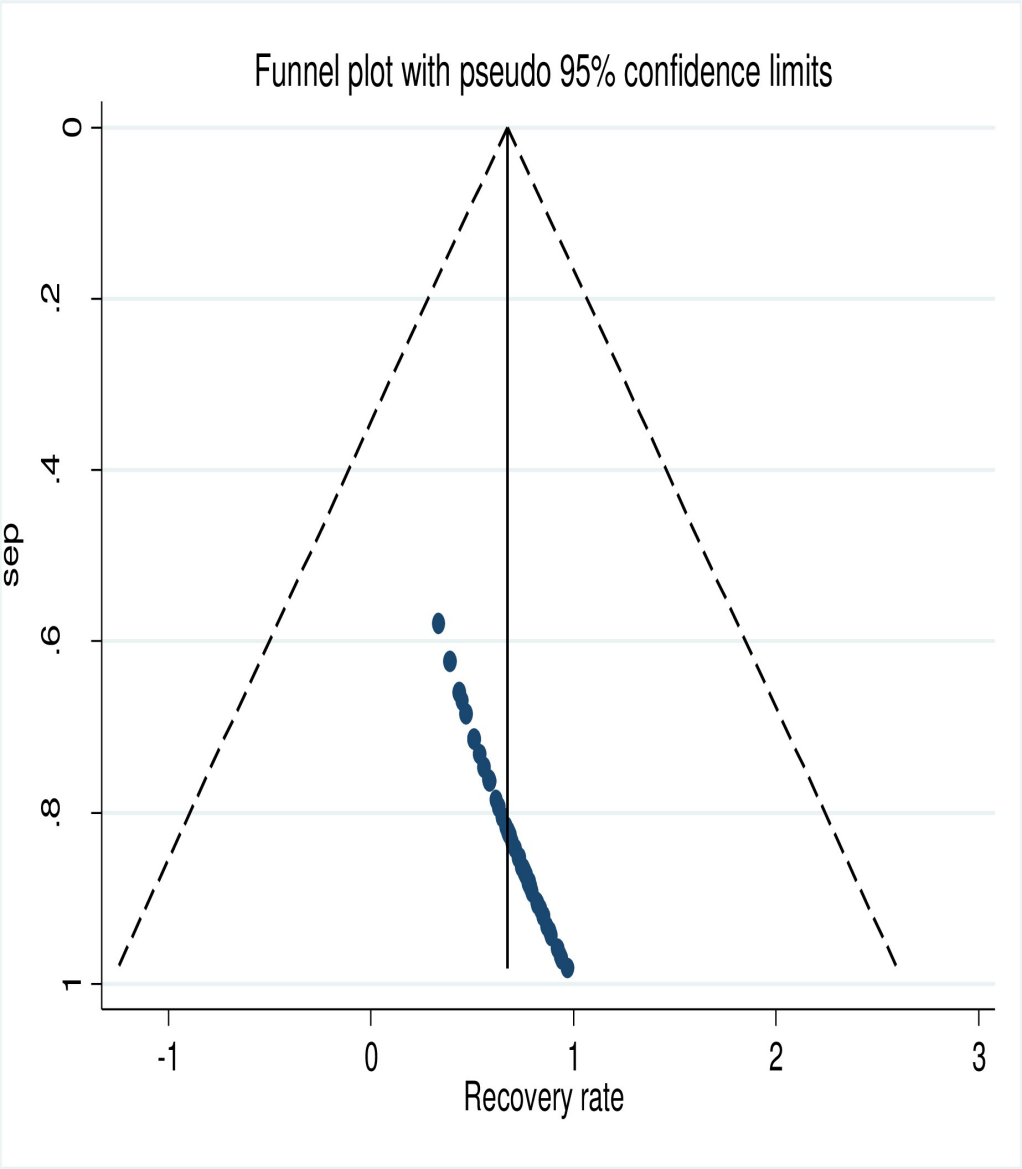
Funnel plot presented the visual inspection of publication bias for systematic review and meta-analysis of studies conducted on SAM children, 2019, N = 54, Sub-Saharan Africa.

Publication bias was assessed by visual inspection of funnel plots based on the shape of the graph, symmetrical graph was interpreted to suggest an absence of publication bias whereas an asymmetrical one indicates the presence of publication bias. On the other hand, quantitatively, the Egger’s weighted regression test was used to assess publication bias with p<0.1 considered as indicative of statistically significant publication bias. Since publication bias existed, we did perform Duval and Tweedie's nonparametric 'trim and fill' analysis to formalize the use of funnel plot, estimate number, and outcomes of missed studies, and adjusted for theoretically missed studies [27]. (Fig 1)

### Sensitivity analysis

**Fig 2:**
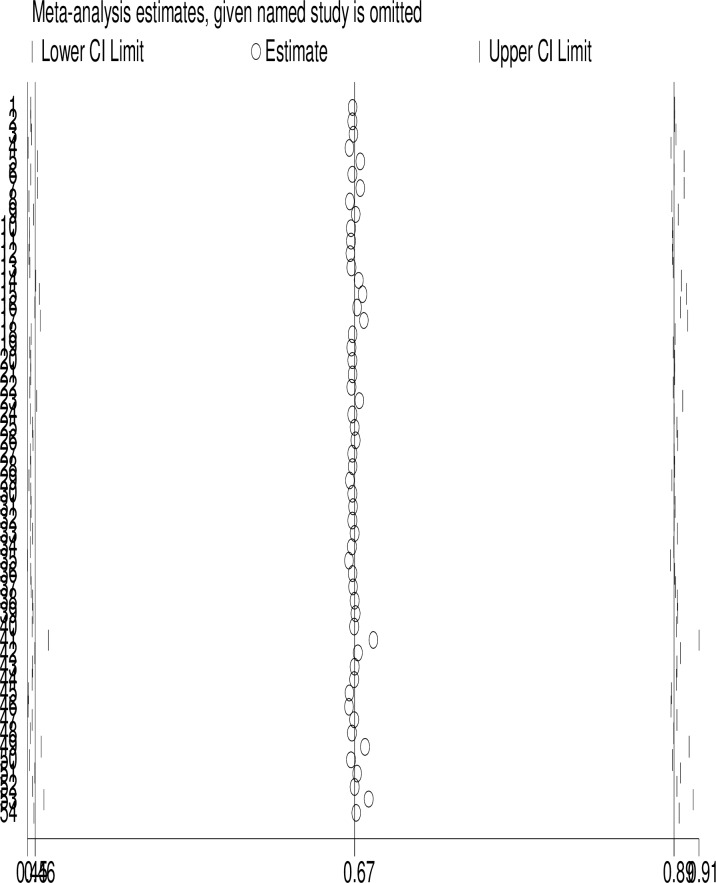
One-leave-out sensitivity analysis for studies conducted on sever acute malnutrition children, 2019, N = 54, Sub-Saharan Africa.

A leave-one-out sensitivity analysis was performed to confirm whether there were studies potentially biased the direction of the pooled estimate. Based on the leave-one-out sensitivity analysis, no outlier study that significantly shifted the primary pooled estimate was found. (Fig 2)

### Statistical analysis

Data were analyzed in Stata V.14 (Stata/IC). Data was presented in the evidence table and summarized using descriptive statistics. The effect measure for SAM recovery rate was computed using the *Metaprop* command for the meta-analysis of proportions in Stata. In this review, the SAM recovery rate was calculated together with its corresponding 95% CI. Besides, estimates for risk factors obtained from each study were pooled and determined as a single estimate. Before analysis, log transformation of odds ratios was performed. A forest plot was generated to show the individual and pooled SAM recovery rate, 95% CI, the author's name, publication year, and study weights.

## Result

### Study screening process

**Fig 3:**
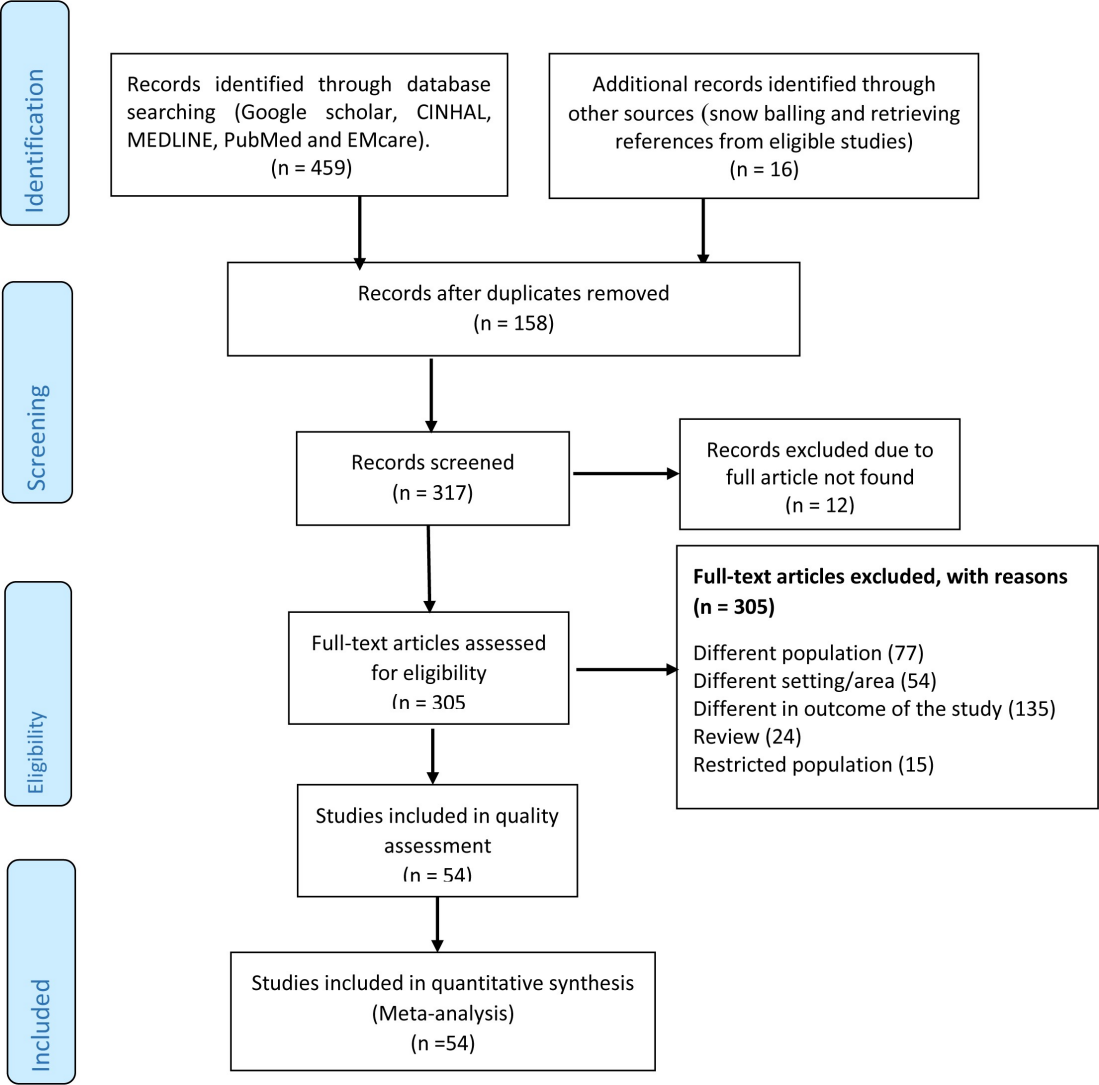
PRISMA statement presentation for meta-analysis of recovery rate among SAM under-five children in Sub-Saharan African countries.

From electronic data basses, we retrieved 475 primary studies in sub-Saharan Africa. After screening their titles and abstracts, 421 unrelated articles were excluded. The full-texts of 54 papers met the inclusion criteria and were included in the final systematic review and meta-analysis (Fig 3). Quality assessment of the studies showed that sixteen studies had fair quality while the rest forty-two studies had good quality. (Table 1)

### Study characteristics

The most retrieved studies (32 studies) were from Ethiopia [4,5,17,18,28–55], followed by Malawi (n = 6 studies) [56–61], and Nigeria (n = 3 studies) [62–64]. Cameroon [65,66], Kenya [67,68], Sudan [69,70], and Ghana [71,72] were represented by two studies, whereas South Africa [73], Uganda [74], Niger [75], Zambia [76], and Democratic Republic of Congo (DRC) [77] were represented by one study. The highest (n = 35 studies) number of reported studies were undertaken at inpatient treatment centers and the rest (n = 18 studies) were at an outpatient treatment program. Similarly, most of the studies were conducted in Hospital setup (n = 32), whereas seven and four studies were at health centers and health post-treatment centers, respectively. The majority (47 studies) of included studies were cross-sectional in design, and the remaining were cohort. Twenty-four and 21 studies used MUAC of 11 cm and 11.5cm as cutoffs, respectively, to define children with SAM. However, a small number (n = 9 studies) of studies did not describe the cutoff point for MUAC. The smallest study was a study by *Attia S et al*. *2016* (included only 79 samples), and the most prominent study was a study by *Chitekwe S et al*. *2018* (included 102,245 samples) in Nigeria and mean sample size for included studies was 2,595.

### Pooled estimates of recovery rate and its determinants

The analysis of fifty-four ranked as fair to good quality studies estimated that the pooled recovery rate of SAM children was 71.2% (95% CI: 68.5–73.8; I^2^ = 98.9%). High heterogeneity was observed among the included studies (Q test p<0.001) and I^2^ (I^2^ = 98.9%). Due to the heterogeneity of included studies, further sub-group analysis was done by using the following study characteristics: country location, admission measurement tools, type of treatment program (inpatient and outpatient), and type of treatment setup (Hospital, Health center, Health post). The random-effect model was applied for reporting a pooled prevalence of the sub-analysis.

On sub-analysis by country location, acceptable SAM recovery rate was observed in the Eastern Africa (PP = 71.4%; 95%CI: 67.4–75.4; n = 41 studies), Central Africa (PP = 84.7%; 95%CI: 68.7–99.7%; n = 3 studies), and Northern Africa (PP = 78.9%; 95%CI: 72.8–85.1; n = 2 studies). Oppositely, the Western (PP = 60.9%; 95%CI: 47.4–74.4; n = 6 studies) and Southern Africa (PP = 64.7%; 95%CI: 43.2–86.2; n = 2 studies) had unacceptable SAM recovery rate. Except in Eastern Africa, the number of studies was too small to interpret in other locations even though the quality of the included studies was acceptable.

Comparable recovery rate was observed among the inpatient (PP = 70.4%, 95% CI: 65.4–75.3; n = 35) and outpatient (PP = 71.1%, 95% CI: 66.2–76.0; n = 18) treatment programs. Likewise, a pooled recovery rate of significant number of studies showed that children had higher recovery rate if treated in Health Centers (PP = 81.3%, 95% CI: 76.2–86.5) and Health Posts (PP = 76.4%, 95% CI: 69.1–83.7) than Hospitals (PP = 69.9%, 95% CI: 65.7–74.1). Slightly greater recovery rate was evidenced among children who were admitted with a MUAC of 11.5cm (PP = 73%, 95% CI: 67.8–78.2; n = 21) as compared to children admitted with a MUAC of 11cm (PP = 71.3%, 95% CI: 66–76.6; n = 24).

Extracted adjusted odds ratios from primary studies were organized into six themes and pooled to identify a predominant risk factors for recovery rate in Sub-saharn Africa. Accordingly, routine medication (Vitamin-A, Folic Acid, Antibiotics, deworming), old age, weight gain, absence of co-morbidity (Anemia, TB, diarrhea, pneumonia, and AFI(acute febrile illness)), HIV, and presence of edema at admission were identified as a predominant risk factors.

**Fig 4:**
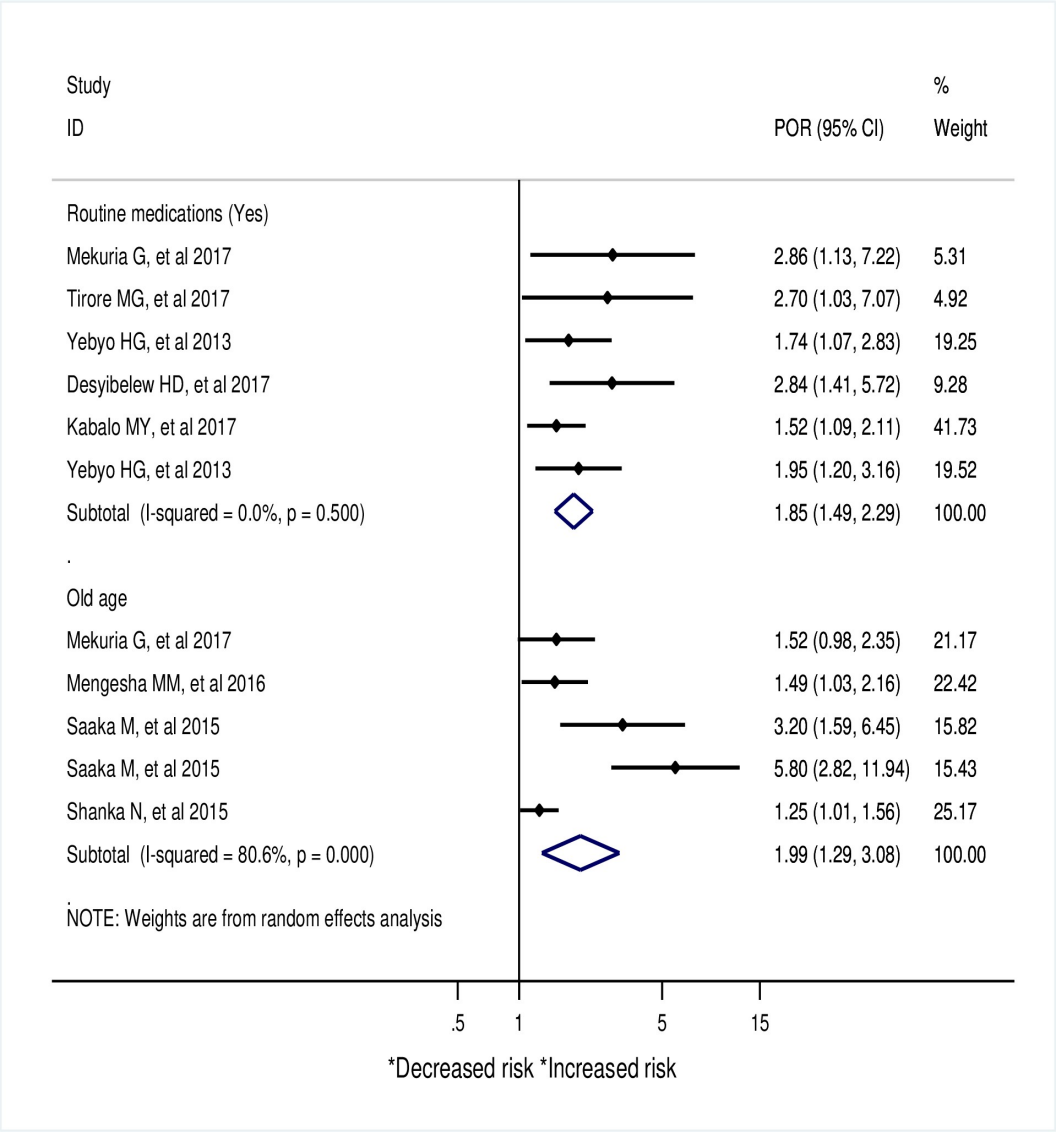
A meta-analysis of factors associated with recovery rate from SAM in Sub-Saharan Africa.

**Fig 5:**
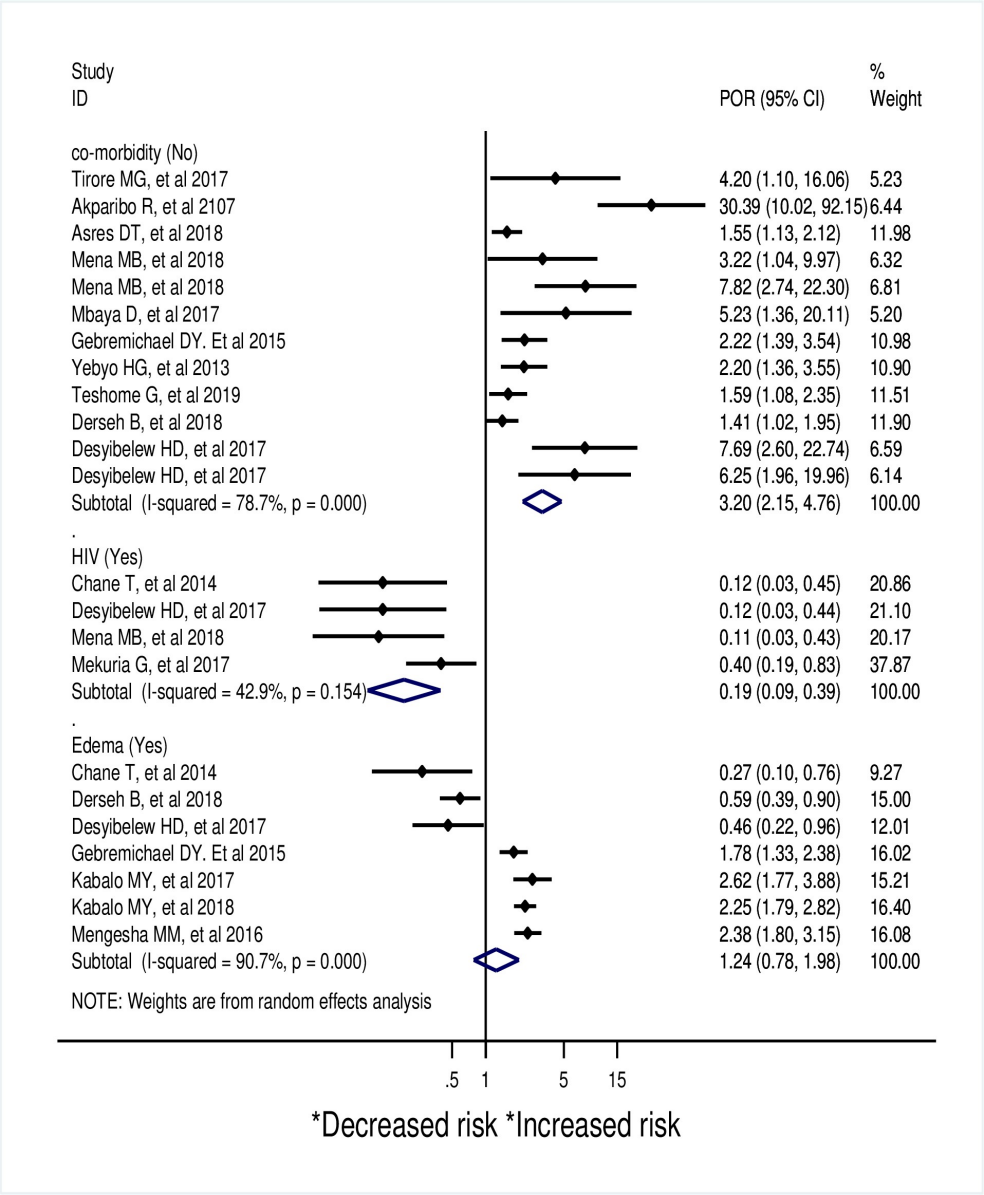
A meta-analysis of factors associated with recovery rate from SAM in under five children in Sub-Saharan Africa.

Accordingly, children who received routine medication (Pooled odds ratio (POR):1.85; 95% CI: 1.49–2.29; *I*^*2*^ = 0.0%), being old in age (POR:1.99; 95% CI: 1.29–3.08; *I*^*2*^ = 80.6%) (Fig 4) and those who did not have co-morbidity (POR: 3.2;95% CI: 2.15–4.76; *I*^*2*^ = 78.7%) had higher odds of recovery. SAM children with HIV infection (POR: 0.19; 95% CI: 0.09–0.39; I^2^ = 42.9%) had lower odds of recovery than their couterparts. (Fig 5)

## Discussion

The current systematic review and meta-analysis has included observational studies published in Sub-Saharan countries and reported the pooled prevalence of children recoverd from SAM admitted to nutritional therapy and the predominant risk factors affected the recovery rate.

The pooled recovery rate of children severely malnourished under-five children admitted to therapy was 71.2%, with marked heterogeneity in the included studies. The pooled recovery rate was below the acceptable cut off point set in SPHERE standard [78]. A bit higher rate of recovery was reported by another systematic review (88.3%) [79], and this high rate of recovery when compared to the current review could be because of majority of the primary studies included in the previos reviews were home-based therapies and were conducted among those children with no complications. Comparable pooled estimates of recovery rate between the inpatient (PP = 70.4%) and outpatient (PP = 71.1%) treatment program was found in the current meta-analysis. However, a similar meta-analysis reporte a better recovery rate (80%) in an outpatient program compared to an inpatient treatment program (71%) [80].

Importantly, we decipher that a significantly high recovery rate was found in Health Centers (PP = 81.3%) and Health Posts (PP = 76.4%) compared to Hospitals (PP = 69.9%). Commonly, uncomplicated SAM children are treated as an outpatient program at health centers and health posts, and on the contrary, SAM children with different infections or co-morbidities are managed at Hospitals, and this could likely reduce their recovery rate [81]. Besides, SAM children treated in Health centers and Health posts usually had a low rate of mortality and default compared to Hospitals, and this also increases their chance of recovery[79]. Based on this meta-analysis, a bit higher recovery rate was observed among children who admitted based on a MUAC threshold of 11.5cm (PP = 73%) as compared to children admitted based on a MUAC threshold of 11cm (PP = 71.3%). As WHO and other meta-analysis evidenced, increasing MUAC cutoffs from 11 cm to 11.5 cm reduces the severity of malnutrition or had a chance of including malnourished children at their early stage that improved their recovery rate [1,81].

The administration of routine medications, in combination with nutritional therapy, has increased the odds of recovery. Routine medications are medications that are given for SAM children according to WHO SAM management guidelines [82]. In support of this, one meta-analysis [80] and other different documents stated that proper adherence to routine medications would have marked positive value for the recovery status [81,83]. Evidence from a randomized, double-blind, placebo-controlled trial [84] and primary studies [45,49] showed that a better recovery rate is associated with an increased age of the children, which is in agreement with the current study. Younger children were indeed less immune and more vulnerable to different infections that play a significant negative role to their recovery. Similar to this meta-analysis finding (POR = 3.2), many meta-analysis and reviews published that the absence of comorbidities significantly increased the odds of recovery rate from SAM [85–87]. Co-morbidities might lead to further complications such as infections, metabolic disturbances, hypothermia, vomiting, severe dehydration, severe anemia, or lack of appetite [81], in which, in combination reduced the odds of recovery rate.

The recovery rate of SAM children with HIV infection was reduced by 81%, which is also published in other meta-analysis findings [86,88] and randomized, double-blind, placebo-controlled trial [84]. SAM children with HIV usually present with multiple opportunistic infections and sepsis with disturbed metabolic status, which complicated the management process, in-turn significantly reduces recovery rate and prolongs treatment time. Even if it was not significant, the recovery rate between edematous and non-edematous children was different, and edematous children had a reasonable low recovery rate than their counters. A review exclusively done in an outpatient program reported a similar result [89].

The finding from the current review might be subject to non-representation of all Sub-saharan countries, and exclusion of non-English based articles might also result in publication bias. So, the interoperation of this finding for further use must account for these inherent limitations of the review.

## Conclusion

The pooled recovery rate from SAM in Sub-Saharan countries was below the acceptable level of SPHERE standard. On the other, Central Africa (PP = 84.7%) and Northern Africa (PP = 78.9%) had a better recovery rate as compared to other regions. According to the current finding, a better recovery rate could be achieved through improving the adherence to the proper administration of routine medications according to SAM management protocol and through careful management of SAM children presented with comorbidities. Furthermore, early detection and treatment of children with SAM would further improve their treatment outcome.

## Supporting information

S1 File (PDF)

S2 File (PDF)

S3 File (PDF)

S4 File (DOCX)

## List of abbreviations

DRC: Democratic Republic of Congo
HIV: Human Immune Virus
MUAC: Mid-Upper-Arm-Circumference
MeSH: Medical Subject Headings
NCHS: National Center for Health Statistics
NOS: Newcastle-Ottawa Scale
OTP: Out-Patient Program
TB: Tuberculosis
WFH: Weight-for-Height
WHZ: weight-for-height Z-score
