# Supplementary material for: The recovery rate from severe acute malnutrition among under-five years of children remains low in sub-Saharan Africa. A systematic review and meta-analysis of observational studies
Source: PLoS One. 2020 Mar 18;15(3):e0229698. doi: 10.1371/journal.pone.0229698 (PMC7080262; doi:10.1371/journal.pone.0229698)
Supplement: S1 File — (PDF) [file pone.0229698.s001.pdf]

Ovid® Wolters Kluwer

My Account | Support & Training | Flinders University Library | Help | Feedback | Logoff

Search | Journals | Books | Multimedia | My Workspace | EBP Tools

▼ **Search History** (27) View Saved

Contract

# ▲	Searches	Results	Type	Actions	Annotations
1	Child Nutrition Disorders/ or Malnutrition/ or Protein-Energy Malnutrition/	21485	Advanced	Display Results | More	
2	Protein-Energy Malnutrition/ or Kwashiorkor/ or Malnutrition/ or Severe Acute Malnutrition/ or Infant Nutrition Disorders/ or Nutritional Status/ or Child Nutrition Disorders/ or Nutrition Disorders/	76366	Advanced	Display Results | More	
3	"severe acute malnutrition".mp. [mp=title, abstract, original title, name of substance word, subject heading word, floating sub-heading word, keyword heading word, protocol supplementary concept word, rare disease supplementary concept word, unique identifier, synonyms]	575	Advanced	Display Results | More	
4	malnutrition.mp. [mp=title, abstract, original title, name of substance word, subject heading word, floating sub-heading word, keyword heading word, protocol supplementary concept word, rare disease supplementary concept word, unique identifier, synonyms]	44504	Advanced	Display Results | More	
5	wasting.mp. [mp=title, abstract, original title, name of substance word, subject heading word, floating sub-heading word, keyword heading word, protocol supplementary concept word, rare disease supplementary concept word, unique identifier, synonyms]	16405	Advanced	Display Results | More	
6	"treatment outcome".mp. or Treatment Outcome/	889213	Advanced	Display Results | More	
7	"recovery rate".mp.	7027	Advanced	Display Results | More	
8	"nutritional recovery rate".mp.	2	Advanced	Display Results | More	
9	treatment outcome.mp. [mp=title, abstract, original title, name of substance word, subject heading word, floating sub-heading word, keyword heading word, protocol supplementary concept word, rare disease supplementary concept word, unique identifier, synonyms]	889213	Advanced	Display Results | More	
10	recovery rate.mp. [mp=title, abstract, original title, name of substance word, subject heading word, floating sub-heading word, keyword heading word, protocol supplementary concept word, rare disease supplementary concept word, unique identifier, synonyms]	7027	Advanced	Display Results | More	
11	1 or 2 or 3 or 4 or 5	107316	Advanced	Display Results | More	
12	6 or 7 or 8 or 9 or 10	895419	Advanced	Display Results | More	
13	11 and 12	3887	Advanced	Display Results | More	
14	Child, Preschool/ or Infant, Newborn/ or Child/ or Infant/	2334351	Advanced	Display Results | More	
15	"under five children".mp. [mp=title, abstract, original title, name of substance word, subject heading word, floating sub-heading word, keyword heading word, protocol supplementary concept word, rare disease supplementary concept word, unique identifier, synonyms]	670	Advanced	Display Results | More	
16	6 to 59 months.mp. [mp=title, abstract, original title, name of substance word, subject heading word, floating sub-heading word, keyword heading word, protocol supplementary concept word, rare disease supplementary concept word, unique identifier, synonyms]	717	Advanced	Display Results | More	
17	14 or 15 or 16	2334729	Advanced	Display Results | More	
18	13 and 17	1059	Advanced	Display Results | More	
19	limit 18 to (english language and humans and last 18 years)	891	Advanced	Display Results | More	
20	"observational study".mp. [mp=title, abstract, original title, name of substance word, subject heading word, floating sub-heading word, keyword heading word, protocol supplementary concept word, rare disease supplementary concept word, unique identifier, synonyms]	101208	Advanced	Display Results | More	
21	case control.mp. [mp=title, abstract, original title, name of	297059	Advanced	Display Results | More	

		substance word, subject heading word, floating sub-heading word, keyword heading word, protocol supplementary concept word, rare disease supplementary concept word, unique identifier, synonyms]			
22	cross-sectional study.mp. [mp=title, abstract, original title, name of substance word, subject heading word, floating sub-heading word, keyword heading word, protocol supplementary concept word, rare disease supplementary concept word, unique identifier, synonyms]	126308	Advanced	Display Results | More	
23	prospective cohort.mp. [mp=title, abstract, original title, name of substance word, subject heading word, floating sub-heading word, keyword heading word, protocol supplementary concept word, rare disease supplementary concept word, unique identifier, synonyms]	49462	Advanced	Display Results | More	
24	retrospective cohort.mp. [mp=title, abstract, original title, name of substance word, subject heading word, floating sub-heading word, keyword heading word, protocol supplementary concept word, rare disease supplementary concept word, unique identifier, synonyms]	48703	Advanced	Display Results | More	
25	retrospective chart review.mp. [mp=title, abstract, original title, name of substance word, subject heading word, floating sub-heading word, keyword heading word, protocol supplementary concept word, rare disease supplementary concept word, unique identifier, synonyms]	21484	Advanced	Display Results | More	
26	20 or 21 or 22 or 23 or 24 or 25	624292	Advanced	Display Results | More	
27	19 and 26	134	Advanced	Display Results | More	

Save Remove Combine with: AND OR

Save All Edit Create RSS View Saved

Basic Search | Find Citation | Search Tools | Search Fields | **Advanced Search** | Multi-Field Search

1 Resource selected | Hide | Change

Ovid MEDLINE(R) and Epub Ahead of Print, In-Process & Other Non-Indexed Citations, Daily and Versions(R) 1946 to December 21, 2018

Enter keyword or phrase (* or $ for truncation)

Keyword Author Title Journal

Search

Limits *(close)* Include Multimedia Map Term to Subject Heading

- Abstracts
- Full Text
- Core Clinical Journals (AIM)
- Structured Abstracts
- Review Articles
- Latest Update
- English Language
- Humans
- Pharmacologic Actions

Publication Year - -

Additional Limits Edit Limits

Options

To search Open Access content on Ovid, go to Basic Search.

Print Email Export + My Projects Keep Selected

Search Information

You searched:

19 and 26

Search terms used:

6
59
acute
case
chart
child
child,
children
cohort
control
cross-sectional

All Range Clear 10 Per Page 1 Go Next ›

1. **Commencing Nutrient Supplements before Full Enteral Feed Volume Achievement Is Beneficial for Moderately Preterm to Late Preterm Low Birth Weight Babies: A Prospective, Observational Study.**

Fan WQ; Gan A; Crane O.

Nutrients. 10(10), 2018 Sep 20.

[Journal Article. Observational Study]

UI: 30241325

Authors Full Name

Fan, Wei Qi; Gan, Amy; Crane, Olivia.

Abstract Reference
Complete Reference

Find Similar
Find Citing Articles

FindIt@Flinders

disorders
five
infant
infant,
kwashiorkor
malnutrition
months
newborn
nutrition
nutritional
observational
outcome
preschool
prospective
protein-energy
rate
recovery
retrospective
review
severe
status
study
to
treatment
under
wasting

Search Returned:
134 text results

Sort By:
-

Customize Display

Filter By

Add to Search History

Selected Only (11)

Years
- **All Years**
- Current year
- Past 3 years
- Past 5 years
- **Specific Year Range**

- **Subject**
- **Author**
- **Journal**
- **Publication Type**

My Projects

+ New Project

No projects available.

JBI EBP Tools

- MANUAL BUILDER
- PAMPHLET BUILDER
- JOURNAL CLUB
- RAPID
- SUMARI

Abstract + My Projects + Annotate

---

2. **Non-Alcoholic Fatty Liver Disease in Overweight Children: Role of Fructose Intake and Dietary Pattern.**

Nier A; Brandt A; Conzelmann IB; Ozel Y; Bergheim I.

Nutrients. 10(9), 2018 Sep 19.

[Journal Article]

UI: 30235828

Authors Full Name
Nier, Anika; Brandt, Annette; Conzelmann, Ina Barbara; Ozel, Yelda; Bergheim, Ina.

Abstract + My Projects + Annotate

Abstract Reference
Complete Reference
Find Similar
Find Citing Articles
FindIt@Flinders

---

3. **Effectiveness of 13-pneumococcal conjugate vaccine (PCV13) against invasive pneumococcal disease in children in the Dominican Republic.**

Tomczyk S; Lessa FC; Sanchez J; Pena C; Fernandez J; Gloria Carvalho M; Pimenta F; Cedano D; Whitney CG; Verani JR; Coradin H; Garib Z; De Oliveira LH; Feris-Iglesias J.

BMC Infectious Diseases. 18(1):152, 2018 04 02.

[Journal Article. Research Support, Non-U.S. Gov't]

UI: 29609548

Authors Full Name
Tomczyk, Sara; Lessa, Fernanda C; Sanchez, Jacqueline; Pena, Chabela; Fernandez, Josefina; Gloria Carvalho, M; Pimenta, Fabiana; Cedano, Doraliza; Whitney, Cynthia G; Verani, Jennifer R; Coradin, Hilma; Garib, Zacarias; De Oliveira, Lucia Helena; Feris-Iglesias, Jesus.

Abstract + My Projects + Annotate

Abstract Reference
Complete Reference
Find Similar
Find Citing Articles
FindIt@Flinders
Full Text

---

4. **Thiopurines are negatively associated with anthropometric parameters in pediatric Crohn's disease.**

Gupta N; Lustig RH; Chao C; Vittinghoff E; Andrews H; Leu CS.

World Journal of Gastroenterology. 24(18):2036-2046, 2018 May 14.

[Journal Article. Observational Study]

UI: 29760546

Authors Full Name
Gupta, Neera; Lustig, Robert H; Chao, Cewin; Vittinghoff, Eric; Andrews, Howard; Leu, Cheng-Shiun.

Abstract + My Projects + Annotate

Abstract Reference
Complete Reference
Find Similar
Find Citing Articles
FindIt@Flinders
Full Text

---

5. **Early Continuous Renal Replacement Therapy Improves Nutrition Delivery in Neonates During Extracorporeal Life Support.**

Murphy HJ; Cahill JB; Twombley KE; Kiger JR.

Journal of Renal Nutrition. 28(1):64-70, 2018 Jan.

[Journal Article]

UI: 28964639

Authors Full Name
Murphy, Heidi J; Cahill, John B; Twombley, Katherine E; Kiger, James R.

Abstract + My Projects + Annotate

Abstract Reference
Complete Reference
Find Similar
Find Citing Articles
FindIt@Flinders

PACES
TAP
CAN-IMPLEMENT

6. **Birthweight, HIV exposure and infant feeding as predictors of malnutrition in Botswanan infants.**

Chalashika P; Essex C; Mellor D; Swift JA; Langley-Evans S.

Journal of Human Nutrition & Dietetics. 30(6):779-790, 2017 Dec.

[Journal Article]

UI: 28960594

Authors Full Name
Chalashika, P; Essex, C; Mellor, D; Swift, J A; Langley-Evans, S.

Abstract + My Projects + Annotate

Abstract Reference
Complete Reference
Find Similar
Find Citing Articles
FindIt@Flinders

---

7. **Air Displacement Plethysmography Versus Bioelectrical Impedance to Determine Body Composition in Pediatric Hemodialysis Patients.**

Wong Vega M; Srivaths PR.

Journal of Renal Nutrition. 27(6):439-444, 2017 Nov.

[Comparative Study. Journal Article]

UI: 28602463

Authors Full Name
Wong Vega, Molly; Srivaths, Poyyapakkam R.

Abstract + My Projects + Annotate

Abstract Reference
Complete Reference
Find Similar
Find Citing Articles
FindIt@Flinders

---

8. **Short versus Extended Duration of Trophic Feeding to Reduce Time to Achieve Full Enteral Feeding in Extremely Preterm Infants: An Observational Study.**

Salas AA; Kabani N; Travers CP; Phillips V; Ambalavanan N; Carlo WA.

Neonatology. 112(3):211-216, 2017.

[Journal Article. Observational Study]

UI: 28704816

Authors Full Name
Salas, Ariel A; Kabani, Nazia; Travers, Colm P; Phillips, Vivien; Ambalavanan, Namasivayam; Carlo, Wally A.

Abstract + My Projects + Annotate

Abstract Reference
Complete Reference
Find Similar
Find Citing Articles
FindIt@Flinders

---

9. **Treating Childhood Malnutrition in Rural Haiti: Program Outcomes and Obstacles.**

Cuneo CN; Dansereau E; Habib AR; Davies M; Ware S; Kornetsky K.

Annals of Global Health. 83(2):300-310, 2017 Mar - Apr.

[Journal Article]

UI: 28619405

Authors Full Name
Cuneo, C Nicholas; Dansereau, Emily; Habib, Anand R; Davies, Mary; Ware, Samuel; Kornetsky, Kenneth.

Abstract + My Projects + Annotate

Abstract Reference
Complete Reference
Find Similar
Find Citing Articles
FindIt@Flinders

---

10. **Beneficial Effect of the Nutritional Support in Children Who Underwent Hematopoietic Stem Cell Transplant.**

Koc N; Gunduz M; Tavil B; Azik MF; Coskun Z; Yardimci H; Uckan D; Tunc B.

Experimental & Clinical Transplantation: Official Journal of the Middle East Society for Organ Transplantation. 15(4):458-462, 2017 Aug.

[Journal Article]

Abstract Reference
Complete Reference
Find Similar
Find Citing Articles

UI: 27765005

FindIt@Flinders

Authors Full Name
Koc, Nevra; Gunduz, Mehmet; Tavil, Betul; Azik, M Fatih; Coskun, Zeynep; Yardimci, Hulya; Uckan, Duygu; Tunc, Bahattin.

Abstract + My Projects + Annotate

All Range Clear 10 Per Page 1 Go Next ›

Print Email Export + My Projects Keep Selected

English Français Italiano Deutsch 日本語 繁體中文 Español 简体中文 한국어

About Us Contact Us Privacy Policy Terms of Use

© 2018 Ovid Technologies, Inc. All rights reserved. OvidSP_UI03.32.00.278, SourceID 117484
