# Supplementary material for: The recovery rate from severe acute malnutrition among under-five years of children remains low in sub-Saharan Africa. A systematic review and meta-analysis of observational studies
Source: PLoS One. 2020 Mar 18;15(3):e0229698. doi: 10.1371/journal.pone.0229698 (PMC7080262; doi:10.1371/journal.pone.0229698)
Supplement: S2 File — (PDF) [file pone.0229698.s002.pdf]

Ovid®

Wolters Kluwer

My Account | Support & Training | Flinders University Library | Help | Feedback | Logoff

Search | Journals | Books | Multimedia | My Workspace | EBP Tools

▼ **Search History** (10)

View Saved

	# ▲	Searches	Results	Type	Actions	Annotations
☐	1	malnutrition/ or recovery rate.mp.	19884	Advanced	Display Results | More	
☐	2	malnutrition/ or malnutrition.mp. or protein calorie malnutrition/	21714	Advanced	Display Results | More	
☐	3	severe acute malnutrition.mp. or malnutrition/	18592	Advanced	Display Results | More	
☐	4	wasting.mp.	4502	Advanced	Display Results | More	
☐	5	1 or 2 or 3 or 4	26498	Advanced	Display Results | More	
☐	6	"nutritional recovery rate".mp. [mp=title, abstract, heading word, drug trade name, original title, device manufacturer, drug manufacturer, device trade name, keyword]	1	Advanced	Display Results | More	
☐	7	"treatment outcome".mp. [mp=title, abstract, heading word, drug trade name, original title, device manufacturer, drug manufacturer, device trade name, keyword]	216536	Advanced	Display Results | More	
☐	8	6 or 7	216537	Advanced	Display Results | More	
☐	9	5 and 8	1083	Advanced	Display Results | More	
☐	10	limit 9 to (human and english and preschool child <1 to 6 years> and last 18 years)	53	Advanced	Display Results | More	

Contract

Save | Remove | Combine with: AND | OR

Save All | Edit | Create RSS | View Saved

Basic Search | Find Citation | Search Tools | Search Fields | **Advanced Search** | Multi-Field Search

1 Resource selected | Hide | Change

Ovid Emcare 1995 to 2018 week 50

Enter keyword or phrase (* or $ for truncation)

◉ **Keyword** ○ Author ○ Title ○ Journal

Search

▾ **Limits** *(close)* ☐ Include Multimedia ☐ Map Term to Subject Heading

☐ Full Text ☐ Latest Update ☐ Abstracts
☐ Human ☐ Cochrane Library ☐ English Language

Publication Year - ▾ - - ▾

EBM-Evidence Based Medicine

-
Evidence Based Medicine
Consensus Development
Meta Analysis
Outcomes Research
Systematic Review

Additional Limits | Edit Limits

Options

To search Open Access content on Ovid, go to Basic Search.

Print | Email | Export | + My Projects | Keep Selected

▼ **Search Information**

You searched:

☐ All | Range | Clear | 10 Per Page ▾ | 51 | Go | ‹ Previous

limit 9 to (human and english and preschool child <1 to 6 years> and last 18 years)

Search terms used:

acute
calorie
malnutrition
nutritional
outcome
protein
rate
recovery
severe
treatment
wasting

Search Returned:
53 text results

Sort By:

-

Customize Display

Filter By

Add to Search History

Selected Only (18)

Years

All Years
Current year
Past 3 years
Past 5 years
Specific Year Range

Subject
Author
Journal
Publication Type

My Projects

+ New Project

No projects available.

JBI EBP Tools

MANUAL BUILDER
PAMPHLET BUILDER
JOURNAL CLUB
RAPID
SUMARI
PACES
TAP
CAN-IMPLEMENT

51. **Sex differences in health indicators among children in African DHS surveys.**

Garenne M.

Journal of Biosocial Science. 35 (4) (pp 601-614), 2003. Date of Publication: October 2003.

[Review]

AN: 37295526

DOI
http://dx.doi.org.ezproxy.flinders.edu.au/10.1017/S00219...

Abstract + My Projects + Annotate

Abstract Reference
Complete Reference
Find Citing Articles
FindIt@Flinders

52. **Implementation of nutrition education and rehabilitation programs (NERPs) in Viet Nam.**

Dickey V.C., Pachon H., Marsh D.R., Lang T.T., Claussenius D.R., Dearden K.A., Ha T.T., Schroeder D.G.

Food and Nutrition Bulletin. 23 (4 SUPP) (pp 78-85), 2002. Date of Publication: December 2002.

[Article]

AN: 36049058

Abstract + My Projects + Annotate

Abstract Reference
Complete Reference
Find Citing Articles
FindIt@Flinders

53. **Role of food antigen elimination in treating children with persistent diarrhea and malnutrition in Zambia.**

Amadi B.

Journal of Pediatric Gastroenterology and Nutrition. 34 (SUPPL. 1) (pp S54-S56), 2002. Date of Publication: 2002.

[Conference Paper]

AN: 34596210

DOI
http://dx.doi.org.ezproxy.flinders.edu.au/10.1097/000051...

Article as PDF (183KB) + My Projects + Annotate

Ovid Full Text
Complete Reference
Find Citing Articles
FindIt@Flinders

All Range Clear 10 Per Page 51 Go ‹ Previous

Print Email Export + My Projects Keep Selected

English Français Italiano Deutsch 日本語 繁體中文 Español 简体中文 한국어

About Us Contact Us Privacy Policy Terms of Use

© 2018 Ovid Technologies, Inc. All rights reserved. OvidSP_UI03.32.00.278, SourceID 117484
